# Label-free LC-MS analysis of HER2+ breast cancer cell line response to HER2 inhibitor treatment

**DOI:** 10.1186/s40199-015-0120-y

**Published:** 2015-08-04

**Authors:** Alessio Di Luca, Michael Henry, Paula Meleady, Robert O’Connor

**Affiliations:** National Institute for Cellular Biotechnology, DCU, Glasnevin, Dublin 9, Dublin, Ireland; School of Nursing and Human Sciences, DCU, Glasnevin, Dublin 9, Dublin, Ireland

**Keywords:** Afatinib, Breast cancer, HER2, Label-free LC-MS Proteomics, Lapatinib, Neratinib

## Abstract

**Background:**

Human epidermal growth-factor receptor (HER)-2 is overexpressed in 25 % of breast-cancers and is associated with an aggressive form of the disease with significantly shortened disease free and overall survival. In recent years, the use of HER2-targeted therapies, monoclonal-antibodies and small molecule tyrosine-kinase inhibitors has significantly improved the clinical outcome for HER2-positive breast-cancer patients. However, only a fraction of HER2-amplified patients will respond to therapy and the use of these treatments is often limited by tumour drug insensitivity or resistance and drug toxicities. Currently there is no way to identify likely responders or rational combinations with the potential to improve HER2-focussed treatment outcome.

**Methods:**

In order to further understand the molecular mechanisms of treatment-response with HER2-inhibitors, we used a highly-optimised and reproducible quantitative label-free LC-MS strategy to characterize the proteomes of HER2-overexpressing breast-cancer cell-lines (SKBR3, BT474 and HCC1954) in response to drug-treatment with HER2-inhibitors (lapatinib, neratinib or afatinib).

**Results:**

Following 12 hours treatment with different HER2-inhibitors in the BT474 cell-line; compared to the untreated cells, 16 proteins changed significantly in abundance following lapatinib treatment (1 μM), 21 proteins changed significantly following neratinib treatment (150 nM) and 38 proteins changed significantly following afatinib treatment (150 nM). Whereas following 24 hours treatment with neratinib (200 nM) 46 proteins changed significantly in abundance in the HCC1954 cell-line and 23 proteins in the SKBR3 cell-line compared to the untreated cells. Analysing the data we found that, proteins like trifunctional-enzyme subunit-alpha, mitochondrial; heterogeneous nuclear ribonucleoprotein-R and lamina-associated polypeptide 2, isoform alpha were up-regulated whereas heat shock cognate 71 kDa protein was down-regulated in 3 or more comparisons.

**Conclusion:**

This proteomic study highlights several proteins that are closely associated with early HER2-inhibitor response and will provide a valuable resource for further investigation of ways to improve efficacy of breast-cancer treatment.

## Background

Breast cancer remains a leading cause of death among women in the Western World. Epidermal growth factor receptor 2 (HER2)-gene amplification or overexpression occurs in approximately 25 % of breast cancers and is associated with poor prognosis [1]. Tyrosine kinases play a critical role in the modulation of growth factor signalling and activated forms of these enzymes plays a crucial role in breast cancer pathogenesis [2]. In spite of the efforts that are underway to develop and improve HER-targeted therapies, *de novo* and acquired resistance remain major obstacles in the clinic; therefore, new drug treatments and methods of accurately predicting drug sensitivity are urgently needed [3].

Lapatinib, neratinib and afatinib are tyrosine kinase inhibitors of HER2 and EGFR (epidermal growth factor receptor) growth factor receptors which prevent the activation of the receptor tyrosine kinase, inhibiting the activation of the pathways that would promote tumour cell growth and proliferation [4]. Lapatinib is an orally active small molecule, it is a first-generation dual tyrosine kinase inhibitors that reversibly binds EGFR and HER2 [5] that has been approved in combination with capecitabine for the treatment of refractory breast cancer [6]. Although lapatinib does not cross the blood–brain barrier, it can reach therapeutic levels in brain tumours and brain metastases [7]. Neratinib and afatinib, two second-generation tyrosine kinase inhibitors that irreversibly bind to multiple HER receptors, are being investigated in clinical trials with promising results either as monotherapy or in combination [8, 9]. Both neratinib and afatinib have the ability to penetrate the blood–brain barrier and, as seen also with lapatinib, these small molecule tyrosine kinase inhibitors have minimal adverse effects on the heart [10].

Proteomics has great potentiality to guide the discovery of biomarkers with clinical utility for the diagnosis, treatment and management of breast cancer. Indeed, the identification of proteins that are differentially expressed as result of exposure to drug treatments such as lapatinib, neratinib and afatinib may provide novel drug targets for improved therapeutic action, and/or predict therapeutic outcome [11]. Mass-spectrometry based proteomics methods, such as label-free LC-MS (liquid chromatography-mass spectrometry), have become more popular for analysing quantitative changes in protein expression between samples [12, 13] though there is a lack of studies investigating the proteomic profile of lapatinib, neratinib or afatinib response in breast cancer.

To identify markers which might be useful in predicting treatment response and/or potential targets for rational additional drug treatments for increasing efficacy, a systematic approach is required. Difficulties in studying hydrophobic proteins or proteins with low or high molecular weights are common inherent proteomic challenges [14]. A method like label-free LC-MS proteomic is ideal for such analyses as it is less impacted by many of these limitations [12, 13]. In this study we have used a quantitative label-free LC-MS proteomic approach to characterize the proteomes of cell line models of HER2-inhibitor response in HER2-positive breast cancer cell lines models, SKBR3, BT474 and HCC1954, in order to further understand the molecular contributors to treatment response.

## Methods

### Cell culture and drug treatment

HER2-overexpressing breast cancer cell lines BT474, SKBR3 and HCC1954 were examined. The BT474 cell line was maintained in antibiotic-free Dulbeccos Modified Eagles medium (DMEM) supplemented with 10 % fetal bovine serum (PAA Labs, Austria), 2 % L-glutamine (Sigma-Aldrich, Germany) and 1 % sodium pyruvate (Sigma-Aldrich, Germany). SKBR3 and HCC1954 breast cancer cell lines were maintained in Roswell Park Memorial Institute (RPMI) 1640 medium supplemented with 10 % fetal bovine serum (PAA Labs, Austria). All cell lines were kept at 37 °C in 5 % CO2/95 % air humidified incubators. Biological replicates, for each cell line, were within 10 passages of each other. All cultures were tested routinely and were mycoplasma-free.

Drug treatments were prepared in dimethyl sulfoxide [(DMSO) Sigma-Aldrich, Germany] at a final concentration of 0.03 % (v/v) and applied as follows lapatinib 1 μM (Sequoia Sciences, Saint Louis, MO, USA), 150 nM afatinib (Sequoia Sciences, Saint Louis, MO, USA) and 150 nM or 200 nM neratinib (Sequoia Sciences, Saint Louis, MO, USA) for 12 or 24 hours of exposure. The drug treatment control consisted of the same cell line treated with the same amount of DMSO used to dilute the drug and for the same exposure time (12 or 24 hours). Three or four biological samples were prepared for each cell lines.

### Protein extraction

Due to the inherent variability of protein fractionation, it was decided to analyse whole cell lysates to minimise differences between the multiple cell lines analysed. After 12 or 24 hours of drug exposure, cells were harvested for LC-MS analysis, following five washes in cold phosphate-buffered saline (PBS) by scraping into cold PBS. Approximately 3 × 10^6^ cells were centrifuged at 200 g and the cell pellet snap frozen in liquid nitrogen.

### Sample preparation for label-free LC-MS analysis

Cell pellets were lysed with lysis buffer {7 M Urea, 2 M Thiourea, 30 mM Tris, 4 % 3-[(3-cholamidopropyl)dimethylamonio]-1-propanesulfonate (CHAPS); pH 8.5} (Sigma-Aldrich, Germany) and then were cleaned up using the Ready Prep 2-D clean up kit (Bio-Rad, Hercules, CA, USA). Protein concentration was determined using the Quick Start Bradford assay (Bio-Rad, Hercules, CA, USA). Ten micrograms of protein sample were resuspended in 40 μl of 50 mM ammonium bicarbonate (Sigma-Aldrich, Germany). Reduction was performed by adding to the samples an amount of dithiothreitol (DTT) (0.5 M) (Sigma-Aldrich, Germany) to reach the concentration of 10 mM at 56 °C for 20 min, and allowed to cool to room temperature. Samples were alkylated by adding to the samples an amount of iodoacetamide (0.55 M) (Sigma-Aldrich, Germany) to reach the concentration of 55 mM and then incubated for 15 min in the dark at room temperature. Digestion with Trypsin Gold, Mass Spectrometry Grade (Promega, Madison, NJ, USA) was carried out at a ratio of 1:19 (Trypsin/Protein) at 37 °C overnight. To stop the digestion, trifluoroacetic acid (TFA) (Sigma-Aldrich, Germany) was add to the samples (0.5 % final concentration) at 37 °C for 15 min. Samples were then cleaned up using Pierce C18 Spin Columns (Thermo Fisher Scientific, USA), dried under a vacuum and stored at −20 °C. Prior to mass spectrometry analysis dried peptides were resuspended in 50 μl of 0.1 % formic acid (FA) in 2 % acetonitrile (ACN) (Sigma-Aldrich, Germany), vortexed to ensure an even suspension.

### Mass spectrometry using LC-MS/MS

Nano LC-MS/MS analysis was carried out using an Ultimate 3000 nanoLC system (Dionex, USA) coupled to a hybrid linear ion trap/Orbitrap mass spectrometer (LTQ Orbitrap XL; Thermo Fisher Scientific, USA). Five μl of digest were loaded onto a C18 trap column (C18 PepMap, 300 m ID × 5 mm, 5 μm particle size, 100 Å pore size; Dionex, USA) and desalted for 10 min using a flow rate of 25 μl/min in 0.1 % TFA in 2 % ACN. The trap column was then switched online with the analytical column [PepMap C18, 75 μm ID × 500 mm, 3 μm particle and 100 Å pore size; (Dionex, USA)] and peptides were eluted with the following binary gradients of solvent A and B: 0–25 % solvent B in 240 min and 25–50 % solvent B in a further 60 min, where solvent A consisted of 2 % ACN and 0.1 % formic acid in water and solvent B consisted of 80 % ACN and 0.08 % formic acid in water. Column flow rate was set to 350 nl/min. Data were acquired in data-dependent mode and externally calibrated with Xcalibur software, version 2.0.7 (Thermo Fisher Scientific, USA). Survey MS scans were acquired in the Orbitrap in the 400–1800 m/z (mass-to-charge ratio) range with the resolution set to a value of 30000 at m/z 400. Up to three of the most intense multiply charged ions (1+, 2+ and 3+) per scan were collision-induced dissociation (CID) fragmented in the linear ion trap. A dynamic exclusion window was applied within 40 s. All tandem mass spectra were collected using a normalised collision energy of 35 %, an isolation window of 3 m/z, and one microscan.

### Label-free LC-MS quantitative profiling

Label-free LC-MS analysis was carried out using Progenesis QI for proteomics software version 4.1 (NonLinear Dynamics, UK), as recommended by the manufacturer (see www.nonlinear.com for further background to alignment, normalisation, calculation of peptide abundance, etc.). As already described by Meleady et al. [15] the software processed the raw data in two steps. Firstly each sample run was subjected to alignment which involved aligning the data based on the LC retention time of each sample; this allows for any drift in retention time giving an adjusted retention time for all runs in the analysis. The sample run that yielded most features (i.e. peptide ions) was used as the reference run, to which retention time of all of the other runs were aligned and peak intensities were normalised. The Progenesis peptide quantification algorithm calculates peptide abundance as the sum of the peak areas within its isotope boundaries. Each abundance value is then transformed to a normalised abundance value by applying a global scaling factor. Protein abundance was calculated as the sum of the abundances of all peptide ions which have been identified as coming from the same protein. A number of criteria were used to filter the data before exporting the MS/MS output files to MASCOT (www.matrixscience.com) for protein identification; peptide features with ANOVA (analysis of variance) p-value ≤0.05 between experimental groups, mass peaks (features) with charge states from +1 to +3, and greater than 3 isotopes per peptide. All MS/MS spectra were exported from Progenesis software as a MASCOT generic file (mgf) and used for peptide identification with MASCOT (version 2.3) searched against the UniProtKB–SwissProt database (taxonomy, *homo sapiens*). The search parameters used were as follows: peptide mass tolerance set to 20 ppm, MS/MS mass tolerance set at 0.6 Da; up to two missed cleavages were allowed, carbamidomethylation set as a fixed modification and methionine oxidation set as a variable modification. Only peptides with ion scores of 40 and above were considered and re-imported back into Progenesis QI for proteomics software for further analysis. A number of criteria were applied to assign a protein as identified; proteins with ≥2 peptides matched, a ≥1.5 fold difference in abundance and an ANOVA between experimental groups of ≤0.05.

The biological function of the proteins identified was assigned using ontology tools in PANTHER [16].

## Results

The different concentrations employed for the three different drug treatments (afatinib, neratinib or lapatinib) were chosen to reflect published clinically relevant concentrations [5, 17, 18]. As the aim of the current study was to investigate proteins that are closely associated with early treatment response and, as a short time (12 hours) exposure to drug treatments may allow identification of a proportionally small number of proteins that are associated with drugs response, another time point (24 hours) and a higher drug concentration were used to ensure that the proteins identified had a higher likelihood of a real association with drug response.

### Proteomic analysis of the HER2-overexpressing breast cancer cell lines

Label-free proteomic analysis was performed in HER2-overexpressing breast cancer cell lines to reveal the proteins that were significantly different in the BT474 cell line in response to afatinib, neratinib or lapatinib treatments and also in the breast cancer cell lines SKBR3 and HCC1954 in response to neratinib treatments. Following analysis of the phenotypes using the software incorporated in Progenesis QI for proteomics, all proteins were ranked by *p* value derived from one-way ANOVA (*p* ≤ 0.05), fold change (≥1.5) and number of peptides (≥2) matched to the protein.

**Fig. 1:**
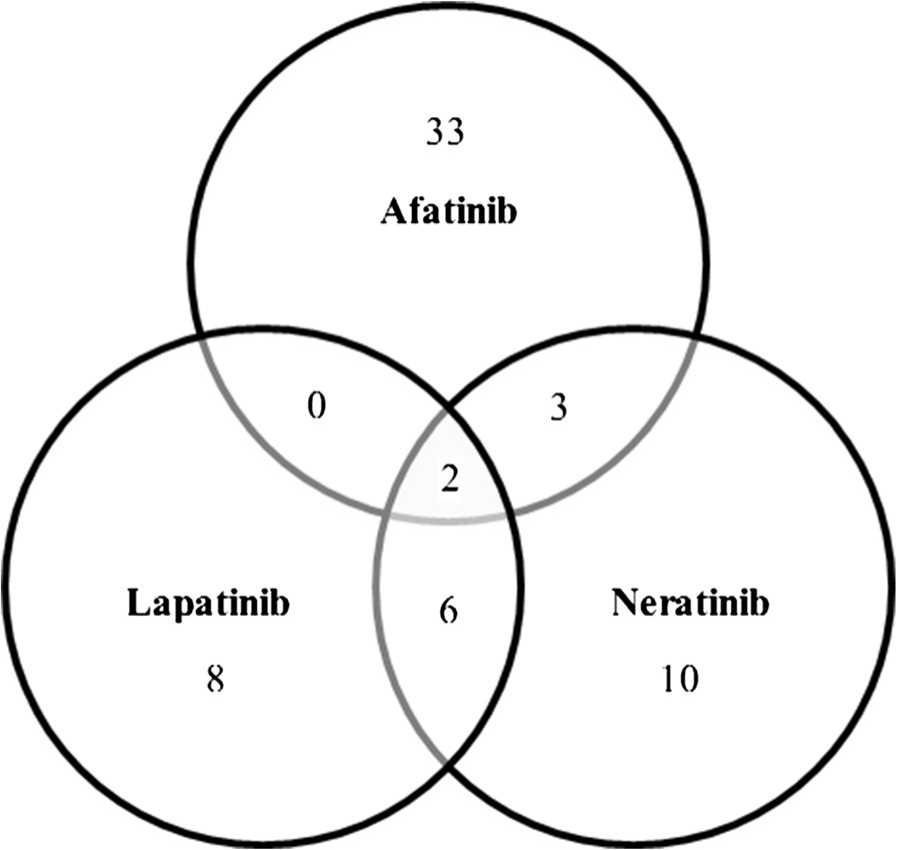
Venn diagrams showing the number of identified proteins with altered levels in response to afatinib, lapatinib or neratinib treatment compared to control in BT474 cell line. The full lists of proteins and the indication if they are up or down regulate are in Tables 1, 2 and 3. Details of the proteins that were in common between different comparisons are in Table 6

38 significant (*p* ≤ 0.05) proteins whose abundance changed significantly in the BT474 between afatinib-treated and control cells were identified. Of these, 16 demonstrated an increased abundance (Table 1a) and 22 a decreased abundance in the treated cells (Table 1b). Details of the significant variation between phenotypes are presented in Table 1. 21 and 16 proteins differed in abundance respectively between the BT474 cell lines neratinib or lapatinib-treated compared to untreated cells (Tables 2 and 3). In the comparison with the neratinib treated cell lines 18 proteins demonstrated an increased abundance (Table 2a) and three a decreased abundance in the treated cells (Table 2b). Of the 16 proteins identified in the comparison with the lapatinib treated cell lines 11 proteins demonstrated an increased abundance (Table 3a) and five a decreased abundance in the treated cells (Table 3b). Figure 1 show a Venn diagram highlighting the proteins that were in common between the proteins that were significantly different in the BT474 cell line in response to afatinib, neratinib or lapatinib treatments. The diagrams shows that 33, ten and eight proteins identified respectively in afatinib, neratinib or lapatinib treated cell line were unique in each comparison. Two proteins were common among all three comparisons, three and six were in common respectively between afatinib and neratinib and between neratinib and lapatinib-treated cells. There were no proteins common between afatinib and lapatinib-treated cells.

**Table 1:** 38 proteins identified as differentially expressed between the afatinib treated BT474 cell line and the control following label-free MS/MS analysis (Progenesis QI for proteomics)

UniProt*^)^	Identification	Peptides^†)^	Score^‡)^	ANOVA (*p*)	Fold change	Highest condition^§)^
a						
O14979;Q14103	Heterogeneous nuclear ribonucleoprotein D-like	4	216.49	0.000367	2.37	Afatinib
Q00839	Heterogeneous nuclear ribonucleoprotein U	3	151.34	0.001436	1.61	Afatinib
P31943	Heterogeneous nuclear ribonucleoprotein H	2	140.67	0.002201	1.58	Afatinib
P40939	Trifunctional enzyme subunit alpha, mitochondrial	2	190.67	0.007201	2.05	Afatinib
Q9Y2X3	Nucleolar protein 58	2	114.49	0.008894	1.86	Afatinib
Q09666	Neuroblast differentiation-associated protein AHNAK	2	90.42	0.008936	1.56	Afatinib
Q8NBS9	Thioredoxin domain-containing protein 5	2	110.22	0.013371	1.56	Afatinib
Q12906	Interleukin enhancer-binding factor 3	2	133.56	0.015709	1.62	Afatinib
P68431	Histone H3.1	2	199.81	0.019319	1.61	Afatinib
P55084	Trifunctional enzyme subunit beta, mitochondrial	1	146.98	0.023507	1.6	Afatinib
O60506	Heterogeneous nuclear ribonucleoprotein Q	1	187.85	0.024443	2.81	Afatinib
P10809	60 kDa heat shock protein, mitochondrial	2	132.64	0.026205	5.23	Afatinib
P09429	High mobility group protein B1	1	140.79	0.027432	1.58	Afatinib
P62937	Peptidyl-prolyl cis-trans isomerase A	3	193.48	0.034894	1.75	Afatinib
A6NMY6	Putative annexin A2-like protein	2	127.79	0.03901	1.69	Afatinib
O43390	Heterogeneous nuclear ribonucleoprotein R	1	217.31	0.049038	2.27	Afatinib
b						
P08133	Annexin A6	2	88.25	0.004506	1.61	Control
A6NEC2	Puromycin-sensitive aminopeptidase-like protein	2	114.96	0.004836	1.75	Control
P48735	Isocitrate dehydrogenase [NADP], mitochondrial	2	100.11	0.005263	1.65	Control
P15531	Nucleoside diphosphate kinase A	2	116.29	0.005349	2.05	Control
P12268	Inosine-5'-monophosphate dehydrogenase 2	2	123.5	0.007421	1.52	Control
P22314	Ubiquitin-like modifier-activating enzyme 1	3	162.89	0.00852	1.58	Control
P00558	Phosphoglycerate kinase 1	3	135.58	0.008782	1.62	Control
P11216	Glycogen phosphorylase, brain form	2	97.04	0.009668	1.67	Control
P61204	ADP-ribosylation factor 3	2	190.14	0.010494	1.64	Control
P50570;Q05193	Dynamin-2	2	115.29	0.011594	1.99	Control
P21796;Q9Y277	Voltage-dependent anion-selective channel protein 1	3	215.59	0.014497	1.58	Control
Q9Y5B9	FACT complex subunit SPT16	2	114.74	0.014521	2.14	Control
P07384	Calpain-1 catalytic subunit	5	261.15	0.015632	1.59	Control
P50395	Rab GDP dissociation inhibitor beta	4	193.14	0.016052	1.55	Control
O43776	Asparagine--tRNA ligase, cytoplasmic	2	84.46	0.019422	1.56	Control
Q14566	DNA replication licensing factor MCM6	3	153.97	0.022003	1.59	Control
P35579	Myosin-9	3	153.68	0.02416	1.68	Control
P49189	4-trimethylaminobutyraldehyde dehydrogenase	2	108.57	0.027635	1.56	Control
Q86VP6	Cullin-associated NEDD8-dissociated protein 1	2	100.91	0.029028	1.67	Control
P04792	Heat shock protein beta-1	3	162.14	0.032666	1.73	Control
P31948	Stress-induced-phosphoprotein 1	2	102.57	0.032815	1.84	Control
Q9NUU7	ATP-dependent RNA helicase DDX19A	2	128.15	0.042435	1.72	Control

16 proteins were up regulated in the cells treated with afatinib (Table 1a) and 22 proteins were up regulated in the untreated cells (control) (Table 1b). ^*)^ Accession number in the UniProt database; ^†)^ Peptides used for quantitation; ^‡)^ MASCOT score. ^§)^ Indicates if the proteins were up regulated in the treated cells (afatinib) or in the not treated cells (control)

**Table 2:** 21 proteins identified as differentially expressed between the neratinib treated BT474 cell line and the control following label-free MS/MS analysis (Progenesis QI for proteomics)

UniProt^*)^	Identification	Peptides^†)^	Score^‡)^	ANOVA (*p*)	Fold change	Highest condition^§)^
a						
P33121	Long-chain-fatty-acid--CoA ligase 1	2	113.74	5.94E-05	1.56	Neratinib
P40939	Trifunctional enzyme subunit alpha, mitochondrial	3	182.37	0.002972	1.51	Neratinib
P0C0S8	Histone H2A type 1	1	138.51	0.003414	3.51	Neratinib
P11021	78 kDa glucose-regulated protein	1	150.58	0.004321	2.87	Neratinib
P78527	DNA-dependent protein kinase catalytic subunit	7	374.79	0.007306	3.89	Neratinib
P0C0S5	Histone H2A.Z	1	151.7	0.007686	2.08	Neratinib
P09874	Poly [ADP-ribose] polymerase 1	5	283.35	0.008246	3.63	Neratinib
P19367	Hexokinase-1	2	130.52	0.009371	1.6	Neratinib
Q12906	Interleukin enhancer-binding factor 3	4	200.23	0.010842	1.59	Neratinib
O43390	Heterogeneous nuclear ribonucleoprotein R	2	116.88	0.011383	1.54	Neratinib
P36542	ATP synthase subunit gamma, mitochondrial	2	95.75	0.011427	1.52	Neratinib
P12956	X-ray repair cross-complementing protein 6	5	275.69	0.014417	1.94	Neratinib
P55084	Trifunctional enzyme subunit beta, mitochondrial	2	99.86	0.015542	1.52	Neratinib
Q99623	Prohibitin-2	2	121.16	0.015667	1.72	Neratinib
P42166	Lamina-associated polypeptide 2, isoform alpha	2	117	0.020737	1.65	Neratinib
P31943	Heterogeneous nuclear ribonucleoprotein H	1	135.7	0.026364	1.61	Neratinib
P80723	Brain acid soluble protein 1	2	112.84	0.029049	3.50	Neratinib
P50416	Carnitine O-palmitoyltransferase 1, liver isoform	2	135.07	0.029912	1.51	Neratinib
b						
Q16643	Drebrin	2	153.59	2.19E-05	1.62	Control
P29966	Myristoylated alanine-rich C-kinase substrate	2	142.02	0.006012	1.59	Control
O95994	Anterior gradient protein 2 homolog	2	199.76	0.011444	1.56	Control

18 proteins were up regulated in the cells treated with neretinib (Table 2a) and three proteins were up regulated in the untreated cells (control) (Table 2a). ^*)^ Accession number in the UniProt database; ^†)^ Peptides used for quantitation; ^‡)^ MASCOT score. ^§)^ Indicates if the proteins were up regulated in the treated cells (neratinib) or in the not treated cells (control)

**Table 3:** 16 proteins identified as differentially expressed between the lapatinib treated BT474 cell line and the control following label-free MS/MS analysis (Progenesis QI for proteomics)

UniProt^*)^	Identification	Peptides^†)^	Score^‡)^	ANOVA (*p*)	Fold change	Highest condition^§)^
a						
P78527	DNA-dependent protein kinase catalytic subunit	2	90.99	0.001596	3.82	Lapatinib
P40939	Trifunctional enzyme subunit alpha, mitochondrial	2	120.25	0.004413	1.66	Lapatinib
O43143	Putative pre-mRNA-splicing factor ATP-dependent RNA helicase DHX15	3	173.99	0.005281	1.57	Lapatinib
P45880	Voltage-dependent anion-selective channel protein 2	2	290.14	0.006264	1.69	Lapatinib
P11021	78 kDa glucose-regulated protein	1	148.58	0.009818	1.94	Lapatinib
P09874	Poly [ADP-ribose] polymerase 1	4	236.21	0.016504	4.24	Lapatinib
P82979	SAP domain-containing ribonucleoprotein	2	150.9	0.020883	1.60	Lapatinib
P42166	Lamina-associated polypeptide 2, isoform alpha	2	127.83	0.026885	1.94	Lapatinib
P62805	Histone H4	3	301.11	0.030621	2.10	Lapatinib
Q92522	Histone H1x	2	136.5	0.037085	1.74	Lapatinib
O43390	Heterogeneous nuclear ribonucleoprotein R	2	111.08	0.048527	1.74	Lapatinib
b						
Q32MZ4	Leucine-rich repeat flightless-interacting protein 1	3	247.18	0.000444	1.62	Control
Q16643	Drebrin	2	149.36	0.001646	1.57	Control
P29966	Myristoylated alanine-rich C-kinase substrate	3	233.58	0.00855	1.64	Control
Q27J81	Inverted formin-2	2	130.65	0.0251	1.79	Control
P04155	Trefoil factor 1	2	126.83	0.026548	1.66	Control

11 proteins were up regulated in the cells treated with lapatinib (Table 3a) and five proteins were up regulated in the untreated cells (control) (Table 3a). ^*)^ Accession number in the UniProt database; ^†)^ Peptides used for quantitation; ^‡)^ MASCOT score. ^§)^ Indicates if the proteins were up regulated in the treated cells (lapatinib) or in the not treated cells (control)

23 proteins were observed to vary significantly (*p* ≤ 0.05) in abundance between the SKBR3 cell line neratinib-treated compared to untreated cells (Table 4). Out of these 23 proteins, 17 demonstrated an increased abundance (Table 4a) and six a decreased abundance in the treated cells (Table 4b).

**Table 4:** 23 proteins identified as differentially expressed between the neratinib treated SKBR3 cell line and the control following label-free MS/MS analysis (Progenesis QI for proteomics)

UniProt^*)^	Identification	Peptides^†)^	Score^‡)^	ANOVA (*p*)	Fold change	Highest condition^§)^
a						
P13489	Ribonuclease inhibitor	4	310.49	0.000561	1.55	Neratinib
Q52LJ0	Protein FAM98B	2	90.26	0.000932	1.62	Neratinib
P49411	Elongation factor Tu, mitochondrial	3	201.31	0.001	1.52	Neratinib
P35232	Prohibitin	2	118.62	0.002224	1.64	Neratinib
Q8WUM4	Programmed cell death 6-interacting protein	2	105.79	0.004641	1.93	Neratinib
P00558	Phosphoglycerate kinase 1	2	201.12	0.00734	1.54	Neratinib
P27348	14-3-3 protein theta	1	151.89	0.008363	1.54	Neratinib
Q00610	Clathrin heavy chain 1	4	233.02	0.00934	1.70	Neratinib
P11177	Pyruvate dehydrogenase E1 component subunit beta, mitochondrial	2	96.98	0.010597	1.73	Neratinib
P50416	Carnitine O-palmitoyltransferase 1, liver isoform	2	126.77	0.010865	1.95	Neratinib
Q00325	Phosphate carrier protein, mitochondrial	2	106.39	0.011586	1.58	Neratinib
Q12931	Heat shock protein 75 kDa, mitochondrial	2	113.29	0.014076	1.77	Neratinib
P31040	Succinate dehydrogenase [ubiquinone] flavoprotein subunit, mitochondrial	2	100.63	0.017419	1.69	Neratinib
P40939	Trifunctional enzyme subunit alpha, mitochondrial	6	423	0.020319	1.71	Neratinib
Q13938	Calcyphosin	3	146.37	0.026178	1.98	Neratinib
P52209	6-phosphogluconate dehydrogenase, decarboxylating	3	152.09	0.035964	4.28	Neratinib
P05091	Aldehyde dehydrogenase, mitochondrial	6	351.3	0.045267	1.80	Neratinib
b						
P11142	Heat shock cognate 71 kDa protein	6	500.28	0.003207	1.59	Control
P06703	Protein S100-A6	2	114.09	0.005483	1.72	Control
P39019	40S ribosomal protein S19	4	213.75	0.005586	1.8	Control
P63220	40S ribosomal protein S21	2	166.6	0.012224	2.43	Control
Q9UNZ2	NSFL1 cofactor p47	2	135.38	0.014751	1.51	Control
P16989	DNA-binding protein A	1	136.69	0.016278	1.53	Control

17 proteins were up regulated in the cells treated with neratinib (Table 4a) and six proteins were up regulated in the untreated cells (control) (Table 4b). ^*)^ Accession number in the UniProt database; ^†)^ Peptides used for quantitation; ^‡)^ MASCOT score. ^§)^ Indicates if the proteins were up regulated in the treated cells (neratinib) or in the not treated cells (control)

46 proteins were observed to vary significantly (*p* ≤ 0.05) in abundance between the HCC1954 cell line neratinib-treated compared to untreated cells (Table 5). Of these, 22 demonstrated an increased abundance (Table 5a) and 24 a decreased abundance in the treated cells (Table 5b).

**Table 5:** 46 proteins identified as differentially expressed between the neratinib treated HCC1954 cell line and the control following label-free MS/MS analysis (Progenesis QI for proteomics)

UniProt^*)^	Identification	Peptides^†)^	Score^‡)^	ANOVA (*p*)	Fold change	Highest condition^§)^
a						
P23141;Q9UKY3	Liver carboxylesterase 1	5	325.17	1.42E-06	42.37	Neratinib
Q07000	HLA class I histocompatibility antigen, Cw-15 alpha chain	3	177.55	0.000129	2.38	Neratinib
P19971	Thymidine phosphorylase	2	106.97	0.000245	24.47	Neratinib
P42224	Signal transducer and activator of transcription 1-alpha/beta	2	188.79	0.000677	3.17	Neratinib
P42330	Aldo-keto reductase family 1 member C3	1	197.69	0.004702	4.75	Neratinib
P16401	Histone H1.5	2	125.54	0.005291	1.91	Neratinib
Q9P2E9	Ribosome-binding protein 1	3	160.16	0.007391	1.75	Neratinib
P42166	Lamina-associated polypeptide 2, isoform alpha	2	181.9	0.009199	1.81	Neratinib
Q04828;P51857	Aldo-keto reductase family 1 member C1	1	154	0.009747	3.28	Neratinib
P00966	Argininosuccinate synthase	2	86.9	0.010658	3.2	Neratinib
P22314	Ubiquitin-like modifier-activating enzyme 1	11	865.85	0.010735	2.32	Neratinib
O00151	PDZ and LIM domain protein 1	2	125.36	0.011806	1.89	Neratinib
Q7Z406	Myosin-14	2	115.9	0.011847	1.84	Neratinib
P52209	6-phosphogluconate dehydrogenase, decarboxylating	7	460.35	0.012246	2.06	Neratinib
P11413	Glucose-6-phosphate 1-dehydrogenase	3	219.9	0.013282	3.65	Neratinib
P21333	Filamin-A	6	427.66	0.018947	1.90	Neratinib
P30101	Protein disulfide-isomerase A3	2	103.92	0.020008	1.56	Neratinib
O43175	D-3-phosphoglycerate dehydrogenase	2	130.68	0.021376	1.56	Neratinib
Q9BRT3	Migration and invasion enhancer 1	2	101.46	0.021575	1.61	Neratinib
O75874	Isocitrate dehydrogenase [NADP] cytoplasmic	2	141.9	0.027272	1.83	Neratinib
O43707	Alpha-actinin-4	4	261.47	0.035891	1.96	Neratinib
P55327	Tumor protein D52	2	157.9	0.047466	8.1	Neratinib
b						
P40926	Malate dehydrogenase, mitochondrial	4	253.25	0.000516	1.68	Control
P11142	Heat shock cognate 71 kDa protein	4	259.61	0.000586	1.65	Control
Q5VTE0	Putative elongation factor 1-alpha-like 3	2	133.8	0.001037	1.60	Control
O60506	Heterogeneous nuclear ribonucleoprotein Q	3	190.1	0.001168	1.78	Control
P19338	Nucleolin	8	505.63	0.001194	1.7	Control
P53999	Activated RNA polymerase II transcriptional coactivator p15	2	116.09	0.001608	1.54	Control
P63244	Guanine nucleotide-binding protein subunit beta-2-like 1	6	388.91	0.001903	1.68	Control
P04083	Annexin A1	4	237.64	0.00211	1.67	Control
P04406	Glyceraldehyde-3-phosphate dehydrogenase	7	764.51	0.002767	1.75	Control
Q02790	Peptidyl-prolyl cis-trans isomerase FKBP4	4	220.23	0.003155	1.93	Control
P06748	Nucleophosmin	3	269.16	0.003382	1.78	Control
P00558	Phosphoglycerate kinase 1	5	330.19	0.004101	1.51	Control
P05120	Plasminogen activator inhibitor 2	2	125.82	0.004534	23.82	Control
P22234	Multifunctional protein ADE2	3	145.9	0.004729	1.75	Control
Q7KZF4	Staphylococcal nuclease domain-containing protein 1	2	103.98	0.005343	1.62	Control
P17301	Integrin alpha-2	2	123.5	0.006948	6.18	Control
P48643	T-complex protein 1 subunit epsilon	2	88.36	0.008121	1.67	Control
P25705	ATP synthase subunit alpha, mitochondrial	2	100.47	0.008561	1.62	Control
P16070	CD44 antigen	2	117.19	0.010439	3.57	Control
P17987	T-complex protein 1 subunit alpha	2	113.87	0.015235	1.75	Control
P50990	T-complex protein 1 subunit theta	3	157.18	0.018346	2.22	Control
P08238	Heat shock protein HSP 90-beta	2	153.98	0.019861	3.16	Control
P48047	ATP synthase subunit O, mitochondrial	2	94.06	0.033513	2.54	Control
Q9UHD8	Septin-9	2	85.27	0.037332	1.61	Control

22 proteins were up regulated in the cells treated with neratinib (Table 5a) and 24 proteins were up regulated in the untreated cells (control) (Table 5b). ^*)^ Accession number in the UniProt database; ^†)^ Peptides used for quantitation; ^‡)^ MASCOT score. ^§)^ Indicates if the proteins were up regulated in the treated cells (neratinib) or in the not treated cells (control)

**Fig. 2:**
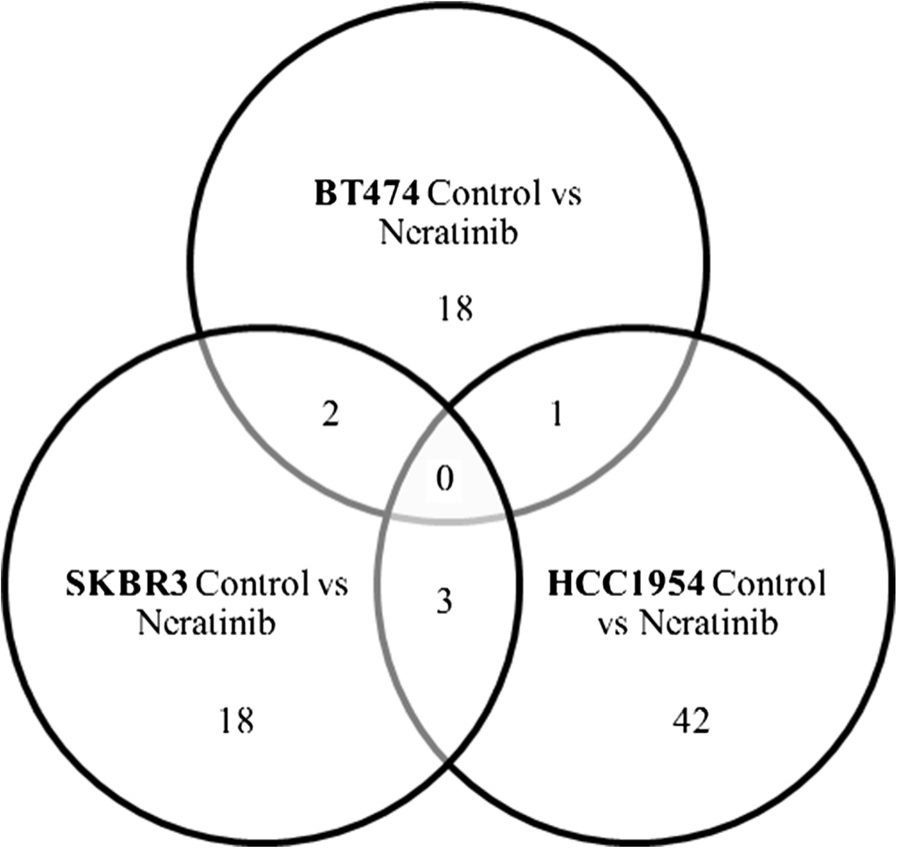
Venn diagrams showing the number of identified proteins with altered levels in response to neratinib treatment compared to control in BT474, SKBR3 and HCC1954 cell lines. The full lists of proteins and the indication if they are up or down regulate are in Tables 2, 4 and 5. Details of the proteins that were in common between different comparisons are in Table 6

Figure 2 show a Venn diagram highlighting the proteins that were in common between the proteins that were significantly different in the three different cell lines (BT474, HCC1954 or SKBR3) in response to neratinib treatments. The diagrams shows that 18, 42 and 18 proteins identified respectively in BT474, HCC1954 or SKBR3 treated cell line were unique in each comparison. There were no proteins in common in all three comparisons. One, three and two proteins were in common respectively between BT474 and HCC1954, between HCC1954 and SKBR3 and between BT474 and SKBR3 neratinib treated cell lines.

Following label-free proteomic analysis 194 and 228 proteins were observed to vary significantly (*p* ≤ 0.05) in abundance respectively between SKBR3 and HCC1954 cell lines or between SKBR3 and HCC1954 cell lines both treated with neratinib. 123 were in common between the two comparisons. In the comparison between SKBR3 and HCC1954 cell lines 104 proteins demonstrated an increased abundance and 90 a decreased abundance in HCC1954 cell line. Of the 228 proteins identified in the comparisons between SKBR3 and HCC1954 cell lines both treated with neratinib 105 proteins demonstrated an increased abundance and 123 a decreased abundance in HCC1954 cell line treated with neratinib.

Table 6 shows a list of 17 proteins that were identified in more than one comparisons between the identified proteins with altered levels in response to afatinib, lapatinib or neratinib treatment compared to control in BT474 cell line and also in neratinib treatment compared to control in HCC1954 and SKBR3 cell lines. There were no proteins in common in all five comparisons. Trifunctional enzyme subunit alpha, mitochondrial was in common and up-regulated among four comparison whereas, heterogeneous nuclear ribonucleoprotein R and lamina-associated polypeptide 2, isoform alpha were in common and up-regulated between three comparisons. 11 proteins are in common between two comparisons, of these eight were up-regulated and three down-regulated among comparisons. Moreover, three proteins showed an irregular abundance pattern in response to different drugs treatment and/or cell line, phosphoglycerate kinase 1 was down-regulated in response to afatinib in BT474 cell line and in response to neratinib in HCC1954 cell line, but was up-regulated in response to neratinib in SKBR3 cell line. Ubiquitin-like modifier-activating enzyme 1 and heterogeneous nuclear ribonucleoprotein Q showed both an opposite abundance pattern in response to afatinib in BT474 cell line and in response to neratinib in HCC1954 cell line. Details are presented in Table 6.

**Table 6:** Proteins differentially expressed that have been identified in more than one comparisons between the identified proteins with altered levels in response to afatinib, lapatinib or neratinib treatment compared to control in BT474 cell line and in neratinib treatment compared to control in HCC1954 and SKBR3 cell lines

Protein	Biological process^a)^	BT474 afatinib vs untreated	BT474 neratinib vs untreated	BT474 lapatinib vs untreated	SKBR3 neratinib vs untreated	HCC1954 neratinib vs untreated
Trifunctional enzyme subunit alpha, mitochondrial	fatty acid metabolism; carbohydrate metabolic process	↑	↑	↑	↑	-
Heterogeneous nuclear ribonucleoprotein R	DNA replication; RNA splicing; mRNA splicing; protein metabolic process	↑	↑	↑	-	-
Lamina-associated polypeptide 2, isoform alpha	immune system process; cellular defense response	-	↑	↑	-	↑
Phosphoglycerate kinase 1	Glycolysis	↓	-	-	↑	↓
Heterogeneous nuclear ribonucleoprotein H	mRNA splicing	↑	↑	-	-	-
Ubiquitin-like modifier-activating enzyme 1	coenzyme metabolic process; cellular protein modification process; proteolysis; cell communication; intracellular protein transport; nuclear transport	↓	-	-	-	↑
Interleukin enhancer-binding factor 3	apoptotic process; purine nucleobase metabolic process; protein metabolic process; cell cycle; neurological system process; response to stimulus; RNA localization	↑	↑	-	-	-
Trifunctional enzyme subunit beta, mitochondrial	protein acetylation	↑	↑	-	-	-
Heterogeneous nuclear ribonucleoprotein Q	DNA replication; RNA splicing; protein metabolic process; cell cycle	↑	-	-	-	↓
DNA-dependent protein kinase catalytic subunit	immune system process; induction of apoptosis; DNA repair; DNA recombination; protein phosphorylation; response to stress	-	↑	↑	-	-
Drebrin	cellular process; cellular component morphogenesis	-	↓	↓	-	-
Myristoylated alanine-rich C-kinase substrate	cell communication	-	↓	↓	-	-
78 kDa glucose-regulated protein	protein folding; response to stress; protein complex biogenesis	-	↑	↑	-	-
Poly [ADP-ribose] polymerase 1	DNA repair; protein ADP-ribosylation; response to stress	-	↑	↑	-	-
Carnitine O-palmitoyltransferase 1, liver isoform	cellular amino acid metabolic process; fatty acid metabolic process	-	↑	-	↑	-
Heat shock cognate 71 kDa protein	immune system process; protein folding; response to stress; protein complex biogenesis	-	-	-	↓	↓
6-phosphogluconate dehydrogenase, decarboxylating	pentose-phosphate shunt	-	-	-	↑	↑

The full lists of proteins are in Tables 1, 2, 3, 4 and 5. ^a)^Biological process of the proteins obtained using PANTHER analysis [16]. Arrows indicate if the proteins were up (↑) or down (↓) regulated in the treated cells vs. control

## Discussion

Despite the improvements in diagnosis and treatment of breast cancer, novel and more efficient tools are needed to guide diagnosis and individualise therapy to improve patient-outcomes and overall survival-rates [19]. In this study, we used label-free LC-MS proteomics to identify proteins associated with HER2-inhibitor drugs response in HER2-positive cell lines. To achieve this we characterised the proteomic response of three HER2-targeting tyrosine kinase inhibitors (lapatinib, neratinib and afatinib) in the overexpressing HER2 BT474 cell line. In addition, because of the complexity of breast cancer and to get a broader perspective in multiple breast cancer cell lines, we characterised the proteomic response to neratinib treatment in two other overexpressing HER2 cell lines (HCC1954 and SKBR3). The different concentrations employed for the three different drug treatments represented the typical pharmacokinetic trough concentration that has been reported from patient trials [5, 17, 18]. This approach allowed us to identify a short list of 14 proteins whose expression level alters with a similar abundance patterns between different comparisons (11 up-regulated and three down-regulated) and that appear to be strongly involved in early treatment response.

Breast cancer cell lines have been widely used to investigate different aspect of the disease, generating reproducible and quantifiable results [20], although several problems may arise when using such models. Firstly, protein expression may differ from cell line to cell line within a single organism [21]. In our study, a high number of proteins (194) were shown to change significantly in abundance between SKBR3 and HCC1954 cell lines. Secondly, no single cell line is truly representative of a primary breast cancer, however, when a panel of cell lines are used as a system they can provide powerful information [22]. This study highlighted several proteins (in the three different cell lines) that had altered abundance in response to neratinib treatment, namely, trifunctional enzyme subunit alpha, mitochondrial; lamina-associated polypeptide 2, isoform alpha; carnitine O-palmitoyltransferase 1, liver isoform and 6-phosphogluconate dehydrogenase, decarboxylating. These proteins are generally involved in the fatty acid metabolism and glycolysis and were up-regulated in response to the drug treatment. Heat shock cognate 71 kDa protein that is involved in stress response was down-regulated in response to neratinib treatment. These proteins were in common between BT474 and SKBR3, between BT474 and HCC1954 or between SKBR3 and HCC1954.

In the current study, we also investigated the protein response to lapatinib, neratinib or afatinib, emerging HER2-inhibitor in BT474 cell lines. Interestingly, trifunctional enzyme subunit alpha, mitochondrial and heterogeneous nuclear ribonucleoprotein R were altered in abundance in all treatments, whereas seven proteins were altered in abundance in two drugs treatments (afatinib and neratinib or neratinib and lapatinib). All these proteins were up-regulated in response to the drugs treatment and are generally involved in the fatty acid metabolism, DNA replication and glycolysis. Drebrin and myristoylated alanine-rich C-kinase substrate were down-regulated in response to neratinib and lapatinib treatment, both proteins are involved in cell mobility.

Interestingly, within the 14 highlighted proteins a higher number (11) were up-regulated in response to drug treatments, these proteins are generally involved in the fatty acid metabolism, DNA replication and glycolysis. It is known that cancer cells are different from those of normal cells, showing an increment of aerobic glycolysis, fatty acid synthesis and glutamine metabolism that are needed for proliferation. Cancer cells reprogram their metabolism in order to satisfy their bioenergetic and biosynthetic requirements. Increased aerobic glycolysis, fatty acid synthesis and glutamine metabolism has been linked to therapeutic resistance in cancer [23]. Several studies highlighted that targeting cellular metabolism may improve the response to cancer therapeutics and the combination of chemotherapeutic drugs with cellular metabolism inhibitors may represent a promising strategy to overcome drug resistance in cancer therapy [23, 24]. In the current study, an up-regulation of these proteins was observed following drugs treatments and may be potential targets to enhances therapeutic efficacy or combats drug resistance. The three proteins down-regulated that were identified, are involved in stress response, cell mobility and in different studies have been shown to be up-regulated in tumours of varied origins [25, 26]. In the current study, following drugs treatments a down-regulation of these proteins was observed, this could probably be due to several reasons like alterations in cell morphology, modulation of the immune response, toxicity of certain drugs [25, 26].

Few proteomics studies have been published to date characterising an extensive drugs response in breast cancer. In a previous study from our group [27], 67 proteins showed a significant change in abundance in response to lapatinib in SKBR3 cell line. Of these, inverted formin-2 was identified in our study in the BT474 cell line treated with lapatinib and was down-regulated in response to drug treatments in both studies, whereas histone H2A type 1 and histone H3.1 were respectively identified as well in the neratinib and afatinib treatment in BT474 cell line with a different abundance pattern between the two studies. Heat shock cognate 71 kDa (HSC70) protein was identified in the neratinib treatment of the SKBR3 and HCC1954 cell lines and was up-regulated in response to drug treatments in both studies.

Of the 17 proteins identified in the current study, some were generally involved in the fatty acid metabolism, of these, as previously mentioned, trifunctional enzyme subunit alpha, mitochondrial was altered in abundance in all three drugs treatments (lapatinib, neratinib or afatinib) in BT474 and in SKBR3 cell line treated with neratinib. Interestingly, the subunit beta was also identified in two comparisons. In all treatments and cell lines both subunit (alpha and beta) identified show a higher abundance in the samples with treatments compared to the control. This is to our knowledge, the first study to report differential abundance of trifunctional enzyme in relation to HER2-positive response breast cancer. This protein is a heterooctamer of four alpha- and four beta-subunits that catalyses three steps in the beta-oxidation spiral of long chain fatty acids. Deficiency of this proteins may cause, vomiting, lethargy, irregular heart rate or sudden, unexpected death [28, 29]. It is known that the use of HER2-targeted monoclonal antibodies like trastuzumab increase the incidence of cardiac dysfunction. A significant reduction of this problem was firstly observed with the use of HER2-targeted small-molecule inhibitors lapatinib and then further decreased incidences evident with the use of neratinib and afatinib second-generation tyrosine kinase inhibitors [30]. The higher abundance of trifunctional enzyme subunit alpha, mitochondrial observed in the current study following the use of novel HER2-targeted therapies may suggest a role for this protein as a marker for testing the toxicity of new HER2-inhibitors.

Heterogeneous nuclear ribonucleoproteins (hnRNPs) are among the most abundant proteins in the eukaryotic nucleus and comprise a family of RNA-binding proteins. These proteins are involved in various steps of messenger RNA (mRNA) biogenesis such as splicing and transport to the cytoplasm [31, 32]. Deregulation of individual hnRNPs was involved in tumour development and progression, including inhibition of apoptosis, angiogenesis and cell invasion [33]. In a study by Chen et al. [34] investigating proteins that are associated with the resistance to paclitaxel in human breast cancer cells, an overexpression of hnRNP C1/C2 was associated with drug resistance. In other studies about lung carcinogenesis a different hnRNP subtype (A2/B1) has been proposed as a marker for early detection [35, 36]. In the current study, different subtypes of this protein (D-like, U, H, Q and R) increased in abundance in one or more drugs treatments (lapatinib, neratinib or afatinib) in BT474 cell line. In particular the R subtypes was identified in all the treatments and may so be useful in predicting an early cellular response in HER2-targetting therapies.

Lamina-associated polypeptide 2, isoform alpha (LAP2α) was up-regulated in two cell lines (BT474 and HCC1954) in response to neratinib and in response to lapatinib in BT474 cell line. LAP2α is one of six splice variants of the mammalian LAP2 gene. LAP2α is a non-membrane protein uniformly distributed throughout the nucleoplasm [37]. Several studies highlighted that this protein may be involved in cancer development or may serve as useful diagnostic and prognostic markers for some types of cancers [38–40]. However, the complex mechanisms of the involvement of this protein in the genesis of the tumour remains to be elucidated, and the contradictory reports in literature regarding its potential role in cell proliferation highlight the need for further work [38, 40]. The up-regulation observed in the current study in the treated cells may provide useful information to predict an early cellular response in HER2-targetting therapies and this information could pave the way to novel strategy to control the development of the cancer.

Proteins involved in the immune system process such as HSC70 were also identified in this study. Stress-related proteins are known as heat-shock proteins (HSP), their role is to protect, preserve or recover the proper functional conformation of proteins, they are divided into families according to their molecular weight. The 70 kDa HSP family is composed of heat inducible proteins (HSP70) that are expressed under cellular stress conditions, and heat shock cognate proteins (HSC70) that are constitutively expressed without any stress stimulation [41]. HSP are overexpressed in patients with malignant tumours, the expression of HSC70 has been reported on breast cancer cells and the overexpression of HSP/HSC70 in chemoresistant cancer cells highlight these proteins as possible clinical markers [25, 42, 43]. In the current study HSC70 was lower in abundance in the cell lines (SKBR3 and HCC1954) treated with neratinib compared to the control. It is known that that the inhibition of HSPs could be related to the toxicity of certain drugs [43] highlighting the chemotherapeutic implications of this protein and the potentiality as a marker to evaluate the potential importance of further treatment options. Similar data were also obtained in response to lapatinib in SKBR3 cell line in a previous study from our group [27].

Taken together, our findings highlight several proteins that are closely associated with early HER2-inhibitor response, complementary studies need to be conducted to validate the importance of our findings and this could have implication for new strategies to improve the efficacy of breast-cancer treatment.

## Conclusion

In conclusion, we have identified several proteins that were differentially expressed following exposure to clinically relevant concentrations of different HER2-inhibitors drug treatments in HER2-overexpressing breast cancer cell lines, lapatinib, neratinib or afatinib in BT474 and in response to neratinib treatment in two other cell lines (HCC1954 and SKBR3). Of these, 14 proteins showed a similar abundance pattern following different drug treatments and/or in different cell lines. In particular, we have identified proteins like trifunctional enzyme subunit alpha, mitochondrial; heterogeneous nuclear ribonucleoprotein R; LAP2α and HSC70 that were altered in abundance in three or more comparisons and may be strongly involved in an early treatment response to HER2-inhibition. These may warrant further investigation for example applying siRNA knockdown protocols to reduce the protein levels in cells and to study the functional consequences of their removal, this likely could have implication for improve efficacy of HER2-inhibitor based breast-cancer treatment.
